# Supplementary material for: Analysis of PPARγ Signaling Activity in Psoriasis
Source: Int J Mol Sci. 2021 Aug 10;22(16):8603. doi: 10.3390/ijms22168603 (PMC8395241; doi:10.3390/ijms22168603)
Supplement: Supplementary file 1 [file ijms-22-08603-s001.zip › Supplemental materials_Analysis of PPARg signaling activity in psoriasis/Pathway models/Models images and html files/PPARG expressed in cells assotiated with psoriasis/236556.html]

Th17 cell


# CellType Th17 cell

|  |  |
| --- | --- |
| URN | urn:agi-ncimcelltype:C2936411 |
| Total Entities | 38 |
| Connectivity | 3141 |
| Name | Th17 cell |

---

|  |  |
| --- | --- |
| ChildConcepts | Th17 cell development |
|  | Th17 cell differentiation |
|  | Th17 cell adhesion |
|  | Th17 cell count |
|  | Th17 cell formation |
|  | Th17 cell migration |
|  | Th17 cell polarity |
|  | Th17 cell proliferation |
|  | Th17 cell phenotype |
|  | Th17 cell population |
|  | Th17 cell function |
|  | Th17 cell survival |
|  | Th17 cell fate commitment |
|  | Th17 cell infiltration |
|  | Th17 cell priming |
|  | Th17 cell homing |
|  | Th17 cell apoptosis |
|  | Th17 cell fate determination |
|  | Th17 cell transendothelial migration |
|  | Th17 cell selection |
|  | Th17 cell fate |
|  | Th17 cell sequestration |
|  | Th17 cell repertoire |
|  | Th17 cell ratio |
|  | Th17 cell growth |
|  | Th17 cell death |
|  | Th17 cell invasion |
|  | Th17 cell division |
|  | Th17 cell homeostasis |
|  | Th17 cell rolling |
|  | Th17 cell localization |
|  | Th17 cell chemotaxis |
|  | Th17 cell distribution |
|  | Th17 cell extravasation |
|  | Th17 cell mediated cytotoxicity |
|  | Th17 cell tethering |
|  | Th17 cell viability |
|  | Th17 cell phagocytosis |

---

|  |  |
| --- | --- |
| Pathway | PPARG expressed in cells assotiated with psoriasis |
|  | Overview. Diseases of the skin (chapter 11) |
|  | 1\_Th2 cell response in asthma |
|  | 2\_3\_Eosinophilia and neutrophilia in asthma: Neutrophil chemotaxis and activation |

---

|  |  |
| --- | --- |
| MedScan ID | 10120772 |

---

|  |  |
| --- | --- |
| Alias | Th17 T cell |
|  | T helper 17 cells |
|  | Th17 cells |
|  | Th17 lymphocyte |
|  | Th17 T lymphocytes |
|  | T helper Th17 cell |
|  | T helper 17 Th17 cells |
|  | Th17 cell |
|  | Th17 T lymphocyte |
|  | helper cell type 17 |
|  | T helper 17 lymphocytes |
|  | T helper 17 lymphocyte |
|  | T helper type 17 |
|  | T helper Th17 cells |
|  | Th17 T cells |
|  | Th17 lymphocytes |
|  | T helper 17 Th17 cell |
|  | T helper 17 cell |

---

|  |  |
| --- | --- |
| NCIm ID | C2936411 |

---
